# Safe Supply at Insite, North America's First Supervised Consumption Site: A Case Report of Reduced Harms From the Toxic Drug Supply in a Client Prescribed Powdered Fentanyl Alternative

**DOI:** 10.1111/dar.70175

**Published:** 2026-05-14

**Authors:** Nadia Fairbairn, Elaine Hu, Mark Lysyshyn, Rupinder Brar, Elizabeth Holliday

**Affiliations:** ^1^ Department of Medicine The University of British Columbia Vancouver Canada; ^2^ Vancouver Coastal Health Authority Vancouver Canada; ^3^ British Columbia Centre on Substance Use Vancouver Canada; ^4^ School of Population and Public Health University of British Columbia Vancouver Canada

## Abstract

The increasingly unpredictable and toxic unregulated drug supply puts people who use drugs at high risk of overdose and death. In April 2022, Insite—North America's first sanctioned supervised consumption site—started a prescribed powdered fentanyl safer supply program available to patients for on‐site consumption. This novel pharmaceutical product, created in partnership with a community compounding pharmacy, can be smoked or injected; is free of dangerous contaminants (e.g., xylazine and non‐prescribed benzodiazepines); and has a predictable concentration. This case describes a man in his 50s with no fixed address and severe opioid use disorder and stimulant use disorder prescribed powdered fentanyl at Insite, which led to reduced use of unregulated drugs, increased supervised consumption site engagement and increased retention on oral opioid agonist therapy. Expansion of supervised consumption services to incorporate the provision of prescribed alternatives to the drug supply for on‐site supervised consumption holds promise and should be evaluated.

## 1 Introduction

Insite—North America's first sanctioned supervised consumption site—opened its doors in Vancouver's Downtown Eastside in 2003 in response to high rates of human immunodeficiency virus (HIV) infection and fatal overdoses. It was established to provide a safe, health‐focused space for people to consume pre‐obtained unregulated drugs [1, 2]. At Insite, service users can access harm reduction supplies (e.g., sterile injection equipment), and staff are present to provide emergency overdose responses, clinical care (e.g., wound care) and referrals to substance use and other health services. Since its establishment, Insite has undergone rigorous evaluation that has contributed high‐quality evidence showing that supervised consumption sites prevent fatal overdoses, reduce infectious disease risk and improve treatment uptake without increasing drug use or crime [3, 4].

As the unpredictable and toxic unregulated drug supply continues to drive an unprecedented number of overdose deaths, emerging evidence indicates that prescribing a safer supply may improve measurable health and well‐being outcomes, including reduced substance use‐related harms, improved physical and mental health and increased quality of life [5, 6, 7]. Prescribed safer supply began operating at a limited number of sites in Ontario in 2016 [8, 9] and was rapidly scaled up across Canada during the COVID‐19 pandemic, with 60 new sites opening nationwide between 1 March and 1 May 2020 alone [10, 11]. While British Columbia has been a key jurisdiction of prescribed safer supply innovation, implementation remains limited, particularly outside of highly medicalised contexts [12].

Beginning in 2022, Insite expanded its suite of harm reduction services to include prescribed safer supply for a limited number of program participants (33 to date, according to internal health authority data), including 14 powdered fentanyl [13]. Required witnesses on‐site limits medication sharing or diversion [14]. We present here the first case, to our knowledge, of offering a fentanyl powder‐prescribed safer supply product within the low‐threshold, harm‐reduction‐oriented setting of a supervised consumption site.

The novel compounded fentanyl powder formulation at Insite is produced in collaboration with a local community compounding pharmacy. Powered fentanyl provides a known and stable potency compared to the irregular concentrations and potencies found in street‐based fentanyl, without contaminants such as benzodiazepines or xylazine [15]. It can be consumed via injection at Insite and is self‐administered by eligible patients with opioid use disorder under healthcare provider supervision. Patients may return to the supervised consumption site for up to four doses per day on an as‐needed basis, regardless of missed doses. Program features include dispensation and supervised consumption of prescribed doses, a requirement to return capsules after use and a post‐consumption observation period in order to reduce diversion risk.

## 2 Case Presentation

A man in his 50s with no fixed address and severe opioid and stimulant use disorders presented to Insite with a history of onset of opioid use disorder following a motor vehicle accident complicated by chronic pain sequelae at age 16, initially involving prescription opioid misuse and later transitioning to injection of unregulated opioids (i.e., heroin) in his twenties. He also had a history of treatment‐naïve chronic hepatitis C infection secondary to injection drug use and post‐traumatic stress disorder from adverse childhood experiences. He reported injecting up to 500 mg (5 ‘points’) of unregulated opioids daily. He reported multiple harms related to substance use, including frequent overdoses and, in the past 2 years, blackouts and seizures from exposure to benzodiazepine‐ and fentanyl‐contaminated unregulated opioids (‘benzo‐dope’). Medications for opioid use disorder, including methadone and injectable opioid agonist therapy (i.e., intravenous hydromorphone), had previously been tried but discontinued as the patient reported them insufficient for managing cravings and withdrawal. He had no prior periods of abstinence or remission from substance use.

The patient had repeat longitudinal clinical assessments at Insite by a nurse practitioner with a speciality fellowship training in addiction medicine. He was first prescribed oral methadone as a first‐line treatment and in accordance with provincial guidelines, though the patient opted to stay on a subtherapeutic dose of 40 mg/day [16]. The nurse practitioner subsequently recommended consideration of prescribed safer supply as part of a comprehensive care plan, and the patient was accordingly counselled regarding access to powdered fentanyl as an off‐label product he could access and consume on‐site at Insite under healthcare supervision. The patient was prescribed doses of 20 mg powdered fentanyl up to a maximum of 80 mg (four doses) per 24 h for self‐administration [17], in addition to a continuation of 40 mg/day of methadone. While an objective of prescribed safe supply was to reduce or replace unregulated opioid use, substances brought into the site were not restricted from consumption under healthcare supervision and in keeping with Insite procedures.

The patient tolerated fentanyl well, with a reduction of opioid withdrawal symptoms and cravings. Over the course of 1 year, he presented to Insite most days and often accessed prescribed fentanyl for all four doses per day and continued to be retained on methadone. Although prescribed fentanyl and methadone did not entirely replace use of unregulated opioids, his use drastically reduced to 50 mg (half a ‘point’) approximately twice per week. After starting the prescribed safer supply program, the patient reported that he consistently consumed any unregulated substances at Insite. He had a total of four overdose events, all at Insite, which were secondary to the consumption of unregulated substances. All events were successfully reversed by the health care staff. He was also supported to successfully access health and dental services and initiated treatment for a hepatitis C infection. The patient still faces socio‐structural barriers to accessing safe and secure housing, which may offer further stabilisation of substance use disorders if obtained.

## 3 Discussion

As the unregulated drug supply has become increasingly unpredictable, potent and contaminated, the resulting morbidity and mortality represent a major public health challenge for people exposed to the toxic drug supply [18, 19]. Opioid doses prescribed to prescribed safer supply clients at Insite far exceed typical MMEs reported in other jurisdictions [20, 21], underscoring the exceptionally high opioid tolerance observed in this setting. These findings highlight both the potential challenges of opioid use disorder treatment retention and the critical role of supervised consumption sites in reducing unregulated drug use‐related harms [2, 3].

Prescribed safer supply represents a novel extension of harm reduction within supervised consumption settings by offering regulated pharmaceutical alternatives to the unregulated drug supply. Evidence from multiple jurisdictions across Canada suggests that prescribed safer supply can reduce overdose mortality among people at the highest risk [22, 23, 24, 25, 26]. In this case, although the patient continued to experience overdoses from unregulated opioids, all events were successfully reversed by on‐site staff. This highlights the importance of combining prescribed safer supply and supervised consumption services for individuals at high risk of overdose. In addition, a prescribed safer supply offered physiologic stability by preventing opioid withdrawal, thereby supporting sustained engagement in care.

The supervised consumption setting further facilitated access to ongoing, essential healthcare services and social supports such as primary care, mental health care and pathways to safe and secure housing within a supportive harm reduction service model. This integrated approach helped bridge the patient to resources offering greater longitudinal stability (in line with the patient's goals) while providing some protection from overdose‐related morbidity and mortality through prescribed safer supply. These findings are particularly relevant in the context of the ongoing safer supply program and supervised consumption site closures in several provinces [27, 28, 29]. Concerns regarding potential harms to individuals and communities in these settings are mitigated through on‐site clinical supervision to reverse and manage drug poisoning events and supervised consumption to reduce the risk of diversion [3, 4, 14].

## 4 Conclusion

This case highlights the opportunity to consider new ways of extending harm reduction services to meet the increasingly complex risk for people who use drugs in the context of the toxic drug supply. Incorporating safer supply initiatives at supervised consumption sites that integrate various harm reduction services and health and social supports may represent a low‐barrier opportunity to engage people in care and reduce harms stemming from the toxic drug supply. Future research and evaluation initiatives are essential to investigate the population‐level impacts of safer supply interventions delivered via supervised consumption sites and longitudinal outcomes, including impact on risk of drug‐related harms, as well as ‘secondary’ benefits of these services through health care engagement and overall improved health and social functioning.

## Author Contributions

Nadia Fairbairn contributed to the conception and design of the work. Elaine Hu and Nadia Fairbairn drafted the manuscript. Elaine Hu, Elizabeth Holliday, Rupinder Brar, Mark Lysyshyn and Nadia Fairbairn critically revised the manuscript for important intellectual content.

## Funding

The authors have nothing to report.

## Consent

The authors have obtained patient consent.

## Conflicts of Interest

Nadia Fairbairn is supported by a Philip Owen Professorship in Addiction Medicine at the University of British Columbia and a Scholar Award from the Michael Smith Foundation for Health Research/St. Paul's Hospital Foundation. The funding organisations had no roles in the project's design, implementation or analysis. The authors declare no conflicts of interest.

## Data Availability

Research data are not shared.
